# Selection of homemade mask materials for preventing transmission of COVID-19: A laboratory study

**DOI:** 10.1371/journal.pone.0240285

**Published:** 2020-10-15

**Authors:** Dijia Wang, Yanjun You, Xiaoli Zhou, Zhiyong Zong, Hao Huang, Hui Zhang, Xin Yong, Yifan Cheng, Liu Yang, Qiong Guo, Youlin Long, Yan Liu, Jin Huang, Liang Du

**Affiliations:** 1 Department of Equipment, West China Hospital, Sichuan University, Chengdu, China; 2 Sichuan Testing Center of Medical Devices, Chengdu, China; 3 Department of Nursing, West China Hospital, Sichuan University, Chengdu, China; 4 Department of Infection Management, West China Hospital, Sichuan University, Chengdu, China; 5 Chinese Evidence-Based Medicine Center, West China Hospital, Sichuan University, Chengdu, China; 6 Emergency Department of West China Hospital, Institute of Disaster Medicine, Department of Emergency and Trauma Nursing, West China Nursing School, Sichuan University, Chengdu, China; 7 West China Hospital, Sichuan University, Chengdu, China; 8 Center for Medical Device Supervision and Evaluation, Chinese Evidence-Based Medicine Center, West China Hospital, Sichuan University, Chengdu, China; Basque Center for Materials, Applications and Nanostructures, PORTUGAL

## Abstract

The Coronavirus Disease 2019 (COVID-19) has swept the whole world with high mortality. Since droplet transmission is the main route of transmission, wearing a mask serves as a crucial preventive measure. However, the virus has spread quite quickly, causing severe mask shortage. Finding alternative materials for homemade masks while ensuring the significant performance indicators will help alleviate the shortage of masks. Referring to the national standard for the “Surgical Mask” of China, 17 materials to be selected for homemade masks were tested in four key indicators: pressure difference, particle filtration efficiency, bacterial filtration efficiency and resistance to surface wetting. Eleven single-layer materials met the standard of pressure difference (≤49 Pa), of which 3 met the standard of resistance to surface wetting (≥3), 1 met the standard of particle filtration efficiency (≥30%), but none met the standard of bacterial filtration efficiency (≥95%). Based on the testing results of single-layer materials, fifteen combinations of paired materials were tested. The results showed that three double-layer materials including double-layer medical non-woven fabric, medical non-woven fabric plus non-woven shopping bag, and medical non-woven fabric plus granular tea towel could meet all the standards of pressure difference, particle filtration efficiency, and resistance to surface wetting, and were close to the standard of the bacterial filtration efficiency. In conclusion, if resources are severely lacking and medical masks cannot be obtained, homemade masks using available materials, based on the results of this study, can minimize the chance of infection to the maximum extent.

## Introduction

In December 2019, the Coronavirus Disease 2019 (COVID-19) outbreak occurred in the city of Wuhan, Hubei province. Up to April 12, 2020, the outbreak has hit all provinces in China and 210 countries across the globe [1], which was declared as a Public Health Emergency of International Concern (PHEIC) by the World Health Organization (WHO) [2]. Droplet transmission is the main routes of COVID-19 transmission. Most guidelines [3–5] recommend the use of masks to prevent droplet transmission, hence wearing a mask is one of the most important preventive measure. MacIntyre et al. [6] showed that adherence to masks significantly reduces the risk of influenza infection (HR = 0.26, 95%CI 0.09–0.77). Brienen et al. [7] showed that population-wide use of face masks could make an important contribution in delaying an influenza pandemic. But during the prediction period in China (from 20 Jan 2020 to 30 Jun 2020), the largest daily facemask shortages were predicted to be 589.5, 49.3, and 37.5 million in each of the three scenarios including the mask-wearing policy in all regions of mainland China, the mask-wearing policy only in Hubei province of China, and the non-implementation of the mask-wearing policy, respectively [8, 9]. Under the current global pandemic situation, the shortage of masks is still severe. Fisher et al. [10], Viscusi et al. [11] have explored methods to alleviate the shortage of masks through reuse after disinfection and prolonged use time. However, with the increase of repeated uses and prolonged use time, the protective effectiveness has significantly reduced [10]. Van der Sande et al. [12] have indicated that the protective factor of surgical masks was 4.1–5.3, while the protective factor of homemade masks was 2.2–2.5, which could reduce the respiratory infections of the population to a certain extent. Davies et al. [13] reported eight kinds of materials such as T-shirts, vacuum cleaner bag, tea cloth and pillowcases, significantly reduced the number of microorganisms expelled, although the surgical mask was three times more effective in blocking transmission than the homemade mask. Therefore, homemade masks using civilian materials is of great value in extreme cases of masks shortage.

The WHO issued an advice on the use of masks in the context of COVID-19 on June 5, 2020, in which mentioned that non-medical masks can be made of different combinations of fabrics, mainly made from various woven and non-woven fabrics, and required at least three layers, depending on the fabric used [14]. The choice of fabric material is different, and the number of material layers required may be different. Factors such as filtration efficiency, breathability, number and combination of materials used, shape, coating and maintenance should be taken into account. Meanwhile, the advice mentioned that there has been few systematic evaluation of these combinations, and for the existing non-medical masks, there are no uniform standards for design, choice of material, layering or shape, and there are also no fixed standards for filtration and breathability [14]. The US Centers for Disease Control and Prevention advised residents to make cloth masks on their own to slow the spread of the virus on April 9 [15], and the National Health Commission for Disease Control issued the “Notice on Printing and Distributing Technical Guidelines for the Selection and Use of Masks for the Prevention of COVID-19 Infections in Different Populations” on February 5, 2020, in which advises home residents, diaspora residents, outdoor activists, and low-risk groups to voluntarily wear masks [16, 17], but lacking guidance on how to select materials scientifically. Inappropriate selection of masks may increase the chance of infection due to failing to play a protective role. Therefore, the study aims to combine the comprehensive literature and expert advice to screen the materials of homemade masks with good accessibility, then through laboratory performance testing, materials suitable for homemade masks are selected to cope with the shortage of medical masks and to protect against respiratory infectious diseases, so as to provide some references for decision-makers.

## Materials and methods

### Selection of homemade mask materials

We searched the PubMed and EMBASE databases systematically and obtained 6 studies [12, 13, 18–21] on civilian homemade mask materials under the epidemic of H5N1 and SARS, including T-shirts, scarves, tea towels, pillowcases, antibacterial pillowcases, vacuum cleaner dust bags, linen, silk, etc. (S1 Table). Then, an expert consensus meeting involved eight experts in related fields including materials (2 people), nursing decontamination (2 people), evidence-based medicine and clinical epidemiology (2 people), and hospital infection management (2 people) was held to determined candidate materials for laboratory testing. Finally, seventeen candidate materials were selected for laboratory testing, including T-shirt, fleece sweater, outdoor jacket, down jacket, sun-protective clothing, jeans, hairy tea towel, granular tea towel, non-woven fabrics shopping bag, vacuum cleaner dust bag, diaper, sanitary pad, non-woven shopping bag, vacuum cleaner bag, pillowcase A (40s × 40s air-jet down-proof fabric), pillowcase B (60s × 60s jet satin), pillowcase C (80s × 60s jet satin), medical non-woven fabric, and medical gauze (S1 File). Furuhashi [22] showed that the disposable mask made of glass fiber mat combined with non-woven fabric proved to be high in performance with a bacterial filtration efficiency of 98.1%-99.4%. As medical device packaging materials, medical non-woven fabrics are widely applied in the field of medical device packaging owing to their high antibacterial properties and strong air permeability [23]. Because it is similar to the material of medical masks, Chinese medical staff used it as a homemade mask material to improve the shortage of masks. Together with experts’ opinions in the relevant fields as well as years of experience, we decided to include it in the study. Medical non-woven fabrics and medical gauze were obtained from the Sterilization and Supply Center of West China Hospital, and the remaining materials were purchased through malls and supermarkets (Table 1).

**Table 1:** Selected candidate materials for homemade masks.

Material	Source	Brand	Fiber composition
T-shirt	Mall	Uniqlo	100% cotton
Fleece sweater	Mall	Uniqlo	100% cotton
Outdoor jacket	Mall	Decathlon	100% polyurethane
Down jacket	Mall	Decathlon	100% polyurethane
Sun-protective clothing	Mall	Decathlon	100% polyester
Jeans	Mall	Uniqlo	98% cotton/2% polyurethane
Hairy tea towel	Supermarket	Maryya	80% polyester/20% nylon
Granular tea towel	Supermarket	Maryya	80% polyester/20% nylon
Non-woven shopping bag	Mall	Eusu	100% polypropylene
Vacuum cleaner bag	Electronic business platform (Jingdong)	Dmy	100% polyethylene-vinyl acetate
Diaper	Supermarket	Elderjoy	Non-woven etc
Sanitary pad	Supermarket	Whisper	Non-woven etc
Pillowcase A	Hospital	Nantong Aokai	40s×40s Air-jet down-proof fabric
Pillowcase B	Hospital	Nantong Aokai	60s × 60s Jet satin
Pillowcase C	Hospital	Nantong Aokai	80s×60s Jet satin
Medical non-woven fabric	Hospital	An Ruiheng	2 Layers of spunbond + 3 layers of meltblown cloth
Medical gauze	Hospital	Rong Wei	Absorbent cotton

### Detection indicator

According to the Chinese standard YY0469-2011 "Surgical Mask" and GB/T4745-2012 "Textiles-Testing and evaluation for water resistance-Spray test method" [24, 25], four key indicators to detect the performance of mask materials were performed, including pressure difference, particle filtration efficiency, bacterial filtration efficiency, and resistance to surface wetting. The definitions and standards of the detection indicators are shown in Table 2 [24, 25]. Chinese standards were formulated on the basis of drawing on international standards combined with its own actual conditions. The results of detection indicators in this study have certain reference value.

**Table 2:** Definitions and standards of detection indicators.

Detection indicator	Definition	Eligibility criteria
Pressure difference /Pa	The resistance of mask with the specified area and specified flow	≤49
Particle filtration efficiency /%	Under specified test conditions, the filter element filters out the percentage of particulate matter	≥30
Bacterial filtration efficiency /%	Percentage filtration of bacteria-containing suspended particles by mask material at a specified flow rate	≥95
Resistance to surface wetting	The resistance of fabrics to wetting or penetration by water, measured by the spray rating	≥3

### Experimental methods

All materials were cut to 18*18 cm, and five samples from each material were tested by Sichuan Testing Center of Medical Devices in China. The pressure difference, particle filtration efficiency and bacterial filtration efficiency were determined by the test method stipulated in the standard of YY0469-2011 "Surgical Masks", and resistance to surface wetting was tested in line with the test method stipulated in the standard of GB/T4745-2012 "Textiles-Testing and evaluation for water resistance-Spray test method". The Qingdao SRP ZR-1200 medical detection instrument on surgical masks was used for pressure difference detection, with the gas flow rate at 8 L/min, the diameter of the sample test zone 25 mm, and the test area 4.9 cm^2^.

The American TSI 8130 automatic filter equipment was employed for testing particle filtration efficiency. The material was first placed in an environment with a relative humidity of 85% and at 38°C for 24 hours for pretreatment and was then sealed in an airtight container. The test was completed within 2 hours after the sample pretreatment. The test process entailed placing the pretreated material in a NaCl aerosol with a relative humidity of 30% and at 25°C (median diameter of particle count 0.075 ± 0.020 μm), with a geometric standard deviation of the particle distribution less than 1.86 and concentration no more than 200 mg/m^3^. The gas flow rate was set to 30 L/min, and the cross-sectional area through which the air flows was 100 cm^2^.

The bacterial filtration efficiency was tested in agreement with the standard of YY0469-2011 "Surgical Mask”. The suspension of Staphylococcus aureus was prepared, followed by sterile plates placed in the A and B chambers of the Qingdao SRP ZR-1000 experimental system, with six layers in each chamber. Chamber A cavity was a positive control, and the pre-treated sample was installed in the cavity B, with the gas flow rate at 28.3 L/min, the bacterial suspension delivery time of the nebulizer 1 minute, and the operation time of sampler 2 minutes. After the test, the tryptic soy peptone agar (TSA) medium plate was incubated at 35°C for 48 hours and removed subsequently. The colony-forming units (positive pores) formed by bacterial particle aerosols were counted afterward, and the number of possible impact particles was converted in accordance with the conversion table [24], and 5 samples were tested using the same method.

The principle of resistance to surface wetting detection was to install the sample on the snap ring and place it at a 45-degree angle to the horizontal, with the center of the sample 150 mm below the nozzle, which was sprayed with 250 mL of distilled water. The spray rating was determined by comparing the appearance of the sample with the evaluation standards and pictures, using Wenzhou Darong Y(B) 813 fabric water-wetting tester as the test instrument.

**Fig 1:**
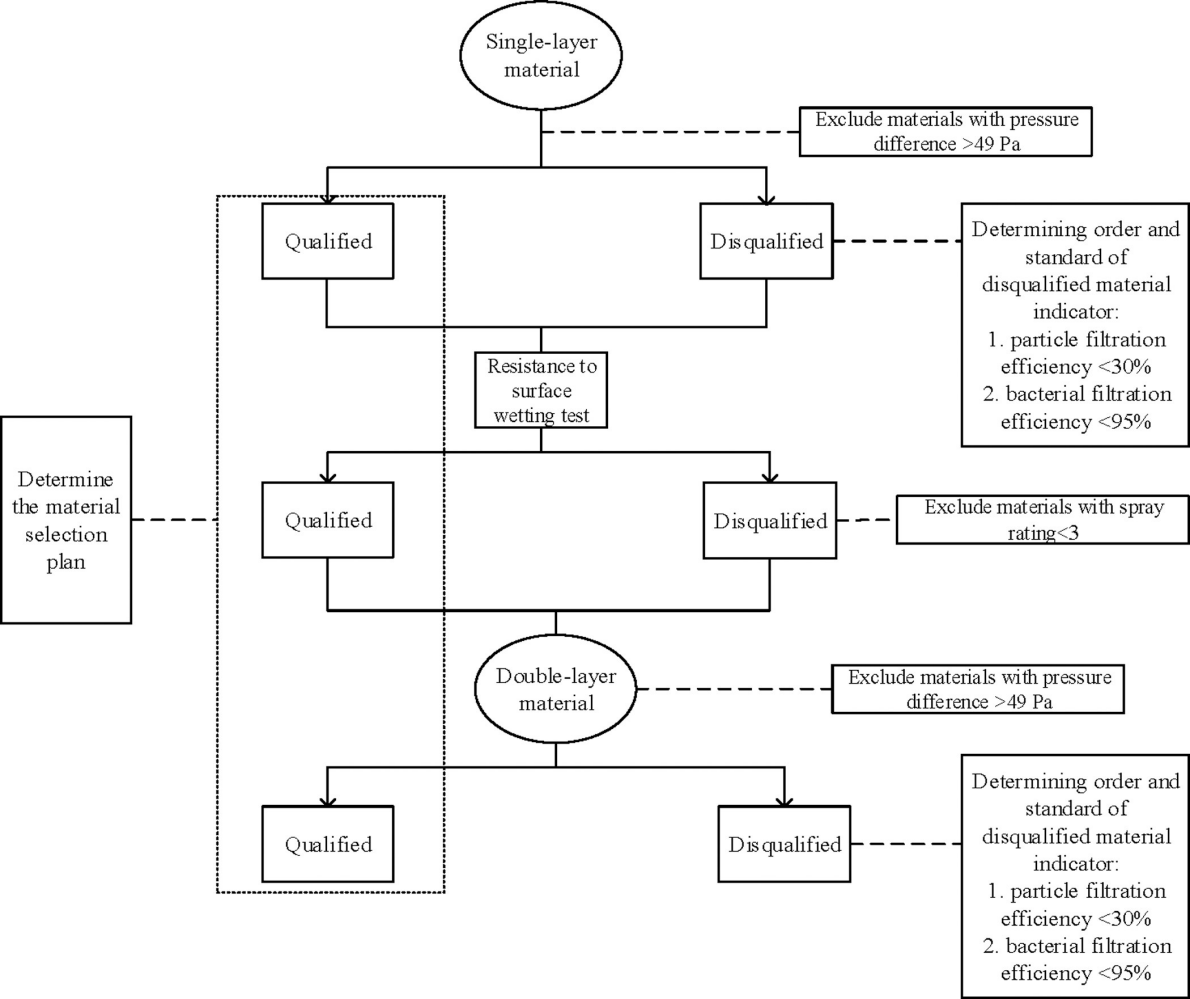


The pressure difference of single-layer materials was firstly tested to exclude materials with a pressure difference over 49 Pa, and the qualified materials of the pressure difference were further tested for particle filtration efficiency, bacterial filtration efficiency and resistance to surface wetting. Because all the single-layer materials had at least one indicator that failed to meet the eligibility criteria, we, based on test results of the single-layer materials, further screened materials with qualified resistance to surface wetting as the outer layer, with the same material or other materials as the inner layer to form double-layer material, to test whether it can meet the standard. Due to the limitation of testing equipment, materials with over two layers cannot be detected thanks to their ultra-thickness, consequently, a combination of multi-layer materials was not further designed (Fig 1).

### Statistical methods

The statistical methods adopted in this study included descriptive analysis, one-sample t test for data with normal distribution, and one-sample Wilcoxon signed-rank test for data with non-normal distribution and grade data. The purpose of this study was to determine whether major indicators of the screened materials are higher than national standards, hence one-sided test was adopted, and the test level was set at one-sided α = 0.05. Since the pressure difference no more than 49Pa was considered above national standards, the null hypotheses of all major indicators except pressure difference indicator are that the screened material level is no less than national standards. The alternative hypothesis is that the screened material level is less than national standards. SPSS18.0 and Microsoft Excel 2016 software were used to perform statistical analysis. The mean, standard deviation, median, t/T value and P value were reported in our study.

## Results

### Test results of homemade mask materials for single-layer

The laboratory testing results showed that 11 materials for single-layer homemade masks had a pressure difference of less than 49 Pa with a P value greater than 0.05, and the order of pressure difference from small to large was as follows: granular tea towel, fleece sweater, medical gauze (4 layers), medical gauze (8 layers), non-woven shopping bag, medical gauze (12 layers), hairy tea towel, T-shirt, medical gauze (16 layers), pillowcase C, and medical non-woven fabrics. The testing results showed that 7 materials for the single-layer homemade mask had a spray rating of more than 3 with a P value great than 0.05. Only three materials met both standards of pressure difference and spray rating, including non-woven shopping bags, medical non-woven fabric, and fleece sweater (Table 3).

**Table 3:** Test results of pressure difference and spray rating for single-layer materials.

Material	pressure difference				Spray Rating			
	$\overset{-}{X}\pm SD$	t/T value	P value	Qualified or Not	Median	T value	P value	Qualified or Not
T-shirt	15.80±1.01	-73.148	1.000	Yes	0	0.000	0.013	No
Fleece sweater	5.86±0.42	-228.642	1.000	Yes	3	3.000	0.922	Yes
Outdoor jacket^a^	-	-	-	No	4	15.000	0.981	Yes
Down jacket	125.10^c^	15.000	0.022	No	4	15.000	0.983	Yes
Sun-protective clothing	125.00^c^	15.000	0.021	No	4	15.000	0.983	Yes
Jeans	124.62±0.99	170.201	<0.001	No	1	0.000	0.019	No
Hairy tea towel	13.72±0.53	-148.556	1.000	Yes	1	0.000	0.017	No
Granular tea towel	5.72±0.13	-742.246	1.000	Yes	1	0.000	0.019	No
Non-woven shopping bag	7.06±0.27	-347.098	1.000	Yes	4	6.000	0.949	Yes
Vacuum cleaner bag^a^	-	-	-	No	4	10.000	0.977	Yes
Diaper	125.44±0.87	196.323	<0.001	No	1	0.000	0.013	No
Sanitary pad	125.44±1.09	156.163	<0.001	No	1	0.000	0.017	No
Pillowcase A	125.34±0.63	268.896	<0.001	No	0	0.000	0.013	No
Pillowcase B	67.80±0.88	47.752	<0.001	No	1	0.000	0.017	No
Pillowcase C	26.86±0.58	-85.791	1.000	Yes	1	0.000	0.017	No
Medical non-woven fabric	35.98±1.85	-15.738	1.000	Yes	4	0.000	0.981	Yes
Medical gauze (4 layers)^b^	6.02±0.64	-150.645	1.000	Yes	-	-	-	No
Medical gauze (8 layers)^b^	6.36±0.42	-225.992	1.000	Yes	-	-	-	No
Medical gauze (12 layers)^b^	8.94±0.50	-178.088	1.000	Yes	-	-	-	No
Medical gauze (16 layers)^b^	17.52±1.33	-52.880	1.000	Yes	-	-	-	No

^a^ The pressure difference of outdoor jacket and vacuum cleaner bag cannot be tested caused by too high ventilation resistance.

^b^ The resistance to surface wetting of medical gauze is not considered, given its main function is to wrap wounds and clean up bloodstains.

^c^ Median.

The particle filtration efficiency test was performed on the materials with qualified pressure difference, and it was found that only the medical non-woven fabric out of the 11 materials had a particle filtration efficiency of over 30% (mean = 42%, SD = 2%, t = 17.789, P>0.05) (Table 4).

**Table 4:** Test results of particle filtration efficiency of materials for single-layer homemade masks with qualified pressure difference.

Material	$\overset{-}{X}\pm SD$	t/T value	P value	Qualified or Not
T-shirt	12%±1%	-34.516	<0.001	No
Fleece sweater	6% ^c^	0.000	0.017	No
Hairy tea towel	23%±1%	-121.000	<0.001	No
Granular tea towel	12%±1%	-13.880	<0.001	No
Non-woven shopping bag	14%±2%	-56.921	<0.001	No
Pillowcase C	0%±0%	-19.124	<0.001	No
Medical non-woven fabric	42%±2%	17.789	1.000	Yes
Medical gauze (4 layers)	2% ^c^	0.000	0.019	No
Medical gauze (8 layers)	3% ^c^	0.000	0.019	No
Medical gauze (12 layers)	6% ^c^	0.000	0.019	No
Medical gauze (16 layers)	14%±1%	-24.422	<0.001	No

^c ^Median.

Further bacterial filtration efficiency testing results of medical non-woven fabric showed that it failed to meet the standard of more than 95% (mean = 62%, SD = 1%, t = -63.934, P<0.05) (Table 5).

**Table 5:** Test results of bacterial filtration efficiency of materials for single-layer homemade masks with qualified pressure difference and particle filtration efficiency.

Material	$\overset{-}{X}\pm SD$	t value	P value	Qualified or Not
Medical non-woven fabric	62%±1%	-63.934	<0.001	No

### Test results of homemade mask materials for double-layer

Fifteen double-layer materials were tested. The results demonstrated that 13 (86.7%) double-layer materials had a pressure difference of less than 49 Pa with a P value greater than 0.05, with double-layer fleece sweater as the minimum pressure difference (mean = 12.40, SD = 1.53, t = -53.501, P>0.05), and medical non-woven fabric plus T-shirt as the maximum pressure difference (mean = 51.06, SD = 1.13, t = 4.091, P<0.05) (Table 6).

**Table 6:** Test results of pressure difference of materials for double-layer homemade masks.

Material	$\overset{-}{X}\pm SD$	t /T value	P value	Qualified or Not
Fleece sweater^d^ +T-shirt^e^	20.32±0.55	-115.743	1.000	Yes
Fleece sweater^d^ + Hairy tea towel^e^	22.84±0.92	-63.522	1.000	Yes
Fleece sweater^d^ + Granular tea towel^e^	14.08±0.83	-94.551	1.000	Yes
Fleece sweater^d^ + Fleece sweater^e^	12.40±1.53	-53.501	1.000	Yes
Non-woven shopping bag^d^ + T-shirt^e^	25.26±1.30	-40.798	1.000	Yes
Non-woven shopping bag^d^ + Hairy tea towel^e^	23.64±1.35	-42.115	1.000	Yes
Non-woven shopping bag^d^ + Granular tea towel^e^	14.44±0.62	-124.063	1.000	Yes
Non-woven shopping bag^d^ + Fleece sweater^e^	14.40±0.77	-100.300	1.000	Yes
Non-woven shopping bag^d^ + Non-woven shopping bag^e^	13.72±0.70	-113.044	1.000	Yes
Medical non-woven fabric^d^ + T-shirt^e^	51.06±1.13	4.091	0.008	No
Medical non-woven fabric^d^ + Fleece sweater^e^	50.90 ^c^	12.000	0.113	Yes
Medical non-woven fabric^d^ + Hairy tea towel^e^	51.00±1.31	3.405	0.014	No
Medical non-woven fabric^d^ + Granular tea towel^e^	43.52±1.48	-8.267	1.000	Yes
Medical non-woven fabric^d^ + Non-woven shopping bag^e^	40.64±1.55	-12.034	1.000	Yes
Medical non-woven fabric^d^ + Medical non-woven fabric^e^	25.66±1.40	-37.155	1.000	Yes

^c^ Median.

^d^ outer material.

^e^ inner material.

Of the 13 double-layer materials which met the standard of pressure difference, nine (69.2%) double-layer material had a particle filtration efficiency of more than 30% with a P value greater than 0.05. The particle filtration efficiency of the fleece sweater plus hairy tea towel was more than 50%, nearly equal to that of double-layer medical non-woven fabric (Table 7).

**Table 7:** Test results of particle filtration efficiency of materials for double-layer homemade masks with qualified pressure difference.

Material^f^	$\overset{-}{X}\pm SD$	t/T value	P value	Qualified or Not
Fleece sweater+ T-shirt	12%±1%	-47.573	<0.001	No
Fleece sweater + Hairy tea towel	56%±1%	58.138	1.000	Yes
Fleece sweater+ Granular tea towel	11%±1%	-42.485	<0.001	No
Fleece sweater + Fleece sweater	11% ^c^	0.000	0.019	No
Non-woven shopping bag+ T-shirt	30%±1%	0.535	0.690	Yes
Non-woven shopping bag+ Hairy tea towel	46% ^c^	15.000	0.981	Yes
Non-woven shopping bag+ Granular tea towel	47%±1%	34.293	1.000	Yes
Non-woven shopping bag + Fleece sweater	35%±2%	6.782	1.000	Yes
Non-woven shopping bag+ Non-woven shopping bag	18% ^c^	15.000	0.018	No
Medical non-woven fabric+ Fleece sweater	35%±1%	9.129	1.000	Yes
Medical non-woven fabric + Granular tea towel	48% ^c^	0.000	0.982	Yes
Medical non-woven fabric + Non-woven shopping bag	40% ^c^	15.000	0.981	Yes
Medical non-woven fabric+ Medical non-woven fabric	54%±1%	63.608	1.000	Yes

^c^ Median.

^f^ The same as the material composition of the inner and outer layers in Table 6.

Concerning the bacterial filtration efficiency, none of the double-layer materials met the standard, but three double-layer materials were close to the standard, including double-layer medical non-woven fabric (mean = 93%, SD = 1, t = -4.000, P<0.05), medical non-woven fabric plus non-woven shopping bag (mean = 89%, SD = 2%, t = -5.477, P<0.05), and medical non-woven fabric plus granular tea towel (mean = 88%, SD = 4%, t = -37.387, P<0.05) (Table 8).

**Table 8:** Test results of bacterial filtration efficiency of materials for double-layer homemade masks with qualified pressure difference and particle filtration efficiency.

Material^f^	$\overset{-}{X}\pm SD$	t value	P value	Qualified or Not
Fleece sweater+ Hairy tea towel	24%±3%	-61.507	<0.001	No
Non-woven shopping bag+ T-shirt	38%±9%	-14.413	<0.001	No
Non-woven shopping bag + Hairy tea towel	23%±2%	-78.071	<0.001	No
Non-woven shopping bag + Granular tea towel	17%±3%	-50.062	<0.001	No
Non-woven shopping bag + Fleece sweater	16%±1%	-135.141	<0.001	No
Medical non-woven fabric+ Fleece sweater	73%±1%	-37.387	<0.001	No
Medical non-woven fabric + Granular tea towel	88%±4%	-4.427	0.006	No
Medical non-woven fabric + Non-woven shopping bag	89%±2%	-5.477	0.003	No
Medical non-woven fabric + Medical non-woven fabric	93%±1%	-4.000	0.008	No

^f^ The same as the material composition of the inner and outer layers in Table 6.

## Discussion

Our study found that the bacterial filtration efficiency of homemade masks failed to meet the standards of surgical masks, but pressure difference and particle filtration efficiency of most materials/material combinations met the standards. For example, the pressure difference and particle filtration efficiency for medical non-woven fabric, both double-layer and single-layer, reached the standard, and the bacterial filtration efficiency of the double-layer medical non-woven fabric was close to the standard of surgical masks.

The medical non-woven fabric in our study is an SMMMS non-woven fabric composed of 2 layers of spunbond and 3 layers of meltblown fabrics. The structure of surgical masks is usually in three-layer: the outer layer is a spunbond nonwoven fabric with a water-blocking effect to prevent droplets from entering the mask; the middle layer is a meltblown nonwoven fabric with a filtering effect; the inner layer is a spunbond nonwoven fabric with the function of absorbing moisture [26]. Among them, meltblown non-woven fabric serves as the most important component [27]. The differences in the process whether electret treatment is performed, weight, and thickness between the two may be the reason why the medical non-woven fabrics are close but fail to meet the standards of surgical masks. The special porous arrangement of medical non-woven fabric enables the steam and other media to penetrate the bag flexibly, which has a significant bacteriostasis effect, and has the characteristics of good breathability, small penetration rate, strong water resistance, and flame retardancy [28]. Li Muping et al. [29] found that the double-layer medical non-woven fabric could effectively block bacteria within 3 months. Zou Xiuzhen et al. [30] discovered that the disposable non-woven fabric material had good antibacterial effectiveness, and was consistent with the results of our study. At present, medical non-woven fabrics are usually supplied directly to hospitals by manufacturers or suppliers. Residents can also purchase them through some e-commerce platforms such as Amazon and Taobao, but their quality assurance has yet to be verified.

Although three fabric materials (T shirt, fleece sweater, and tea towel) tested in our study could not reach the standard of surgical mask in terms of bacterial filtration efficiency, some combinations of the three materials showed a higher level of particle filtration, such as fleece sweater plus hairy tea towel. Studies demonstrated that the filtering performance of fabric materials was similar to surgical masks in some aspects [18, 19]. For example, the permeability of fabric materials such as T-shirts and tea towels under the polydisperse NaCl aerosols was 40% to 90%, while that of a surgical mask was 51% to 89% [18, 19]. Davies et al. [13] reported that tea towels demonstrated high filtration efficiency in both Bacillus atrophaeus and MS2 bacteriophage aerosols, and the filtration efficiency of double-layer tea towels was close to that of medical surgical mask. Van der Sande et al. [12] found that the homemade tea towel mask could still play a protective role to a certain degree and would not be affected by supply restrictions, although its protective effect was not as strong as a surgical mask or FFP2 mask. It is worth noting that elastic materials may be selected for producing T-shirts and fleece sweater, the pore size may increase due to the stretching of the material during testing, which would reduce its filtration efficiency [14]. However, the materials of homemade masks are not fully recognized in available literatures, and their performance indexes were not tested and verified systematically. Based on previous studies and expert opinions, our study included as many homemade mask materials with good accessibility as possible, and tested relevant indicators in strict accordance with national standards. We also explicitly reported whether these materials met the standards, which can provide a more scientific reference for the selection of materials for homemade masks.

Since there are no uniform and clear standards for the performance indicators of homemade masks, and the COVID-19 firstly broke out in Wuhan, China, this study mainly refers to the Chinese "Surgical Masks" standard for experiments under the circumstance of extremely shortage of masks. Whether this study indicators are qualified or not is greatly affected by standards. Certainly, Chinese standards and international standards have similarities and differences, such as the American standard ASTM F2100-11 (2018) and the European standard BS EN 14683:2019 [31]. As for medical surgical masks, the testing procedures and experimental methods included in this study are basically the same as the US and European standards, with the sample testing volume being slightly different [32]. The definitions and requirements of various indicators are not all the same. For example, the definition of pressure difference in China, the United States and Europe is defined as ≤49Pa, <49Pa and <60Pa, respectively; the definition of particle filtration efficiency is defined as ≥30%, ≥98% and lack of clear standard, respectively; and the definition of bacterial filtration efficiency is defined as ≥95%, ≥98% and ≥98%, respectively [32]. Therefore, the experimental results of this study have certain comparability and reference significance.

The purpose of this study is to find homemade mask materials with preferable accessibility and protective performance. When the combination of other materials exceeded 2 layers, the breathability failed to meet the requirements. Therefore, this study only tested single-layer and two-layer material combinations. This does not affect the combination of the preferable three-layer non-medical mask based on the results of this study, because the medical gauze has excellent breathability, the test results of this study showed that the pressure difference of 4-layer medical gauze was only 6.02±0.64, showing that medical gauze has good air permeability. In addition, the medical gauze has good moisture absorption and skin affinity. Based on the test results of the two-layer material combination in this study, when choosing homemade mask material, medical gauze can be used as the inner material, such as using medical non-woven fabric (outer layer) + medical non-woven fabric (middle layer) + medical gauze (inner layer), or medical non-woven fabric (outer layer) + non-woven shopping bag (middle layer) + medical gauze (inner layer) in mask production.

Combining the experimental data and the application target and scope of non-medical masks mentioned in the WHO report, the test materials in this study are not intended to be used by any medical stuff, but for the purpose of source control, this study is expected to provide temporary, accessible, and scientific mouth and nose coverings for the public in and out of public places, such as people at work and gathering when medical masks are indeed scarce to be obtained, so as to play a certain role in respiratory protection. Meanwhile, in addition to wearing a mask, the public should always keep washing hands and practice social distancing [14].

Limitations concerning this study are as follows: the study did not test the flame retardant properties, skin irritation, and delayed-type hypersensitivity of the materials. Samples tested in the study were only the original materials rather than the masks made of these materials. Most of the tested materials were purchased from local supermarkets, thus testing results of these materials could be greatly affected by their types, batches, and manufacturers. The performance of the mask on wearing time, wearing frequency, and environment were not tested because no molded masks were made. All of the data were based on laboratory testing while its actual effectiveness in the protection of the crowd, wearing comfort, adverse reactions still need to be verified by human trials and real-world studies.

## Conclusions

In summary, the study shows that some materials and their combinations for homemade masks could meet several standards of surgical masks. This study is expected to provide temporary, accessible, and scientific mouth and nose coverings for the public in and out of public places. If resources are severely lacking and medical masks cannot be obtained, homemade masks using available materials, based on the results of this study, can minimize the chance of infection to the maximum extent.

## Supporting information

S1 Table Previous research on homemade mask materials. (XLSX)

S1 File Pictures of candidate homemade mask materials. (ZIP)
