# Supplementary figures and images for: Selection of homemade mask materials for preventing transmission of COVID-19: A laboratory study
Source: PLoS One. 2020 Oct 15;15(10):e0240285. doi: 10.1371/journal.pone.0240285 (PMC7561133; doi:10.1371/journal.pone.0240285)

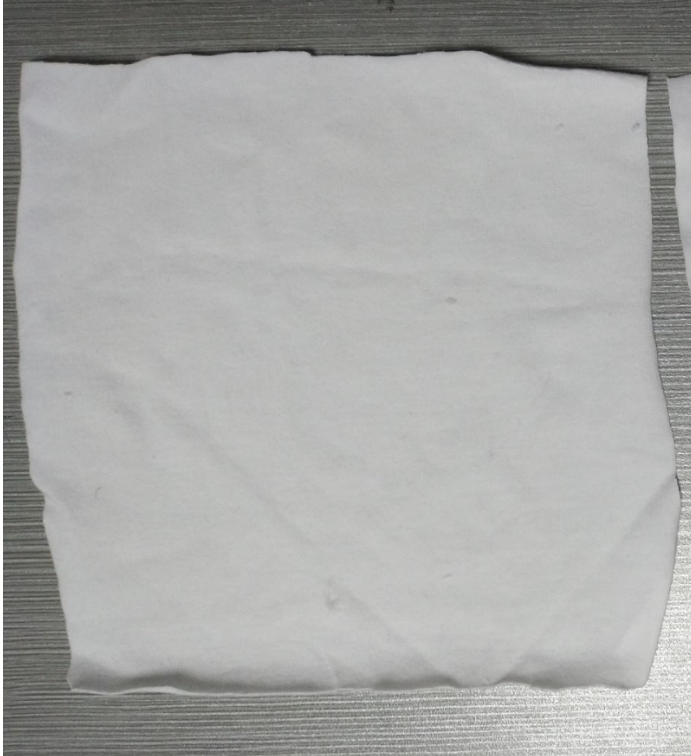

Supplement: S1 File — (ZIP) [file pone.0240285.s002.zip › Pictures of candicate homemade mask materials - ╕▒▒╛/1 T-shirt.PNG]

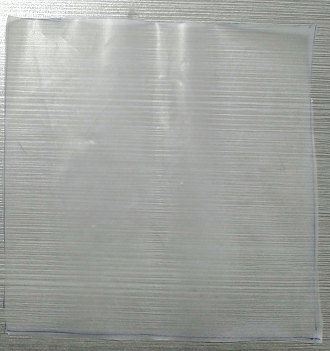

Supplement: S1 File — (ZIP) [file pone.0240285.s002.zip › Pictures of candicate homemade mask materials - ╕▒▒╛/10 Vacuum cleaner bag.PNG]

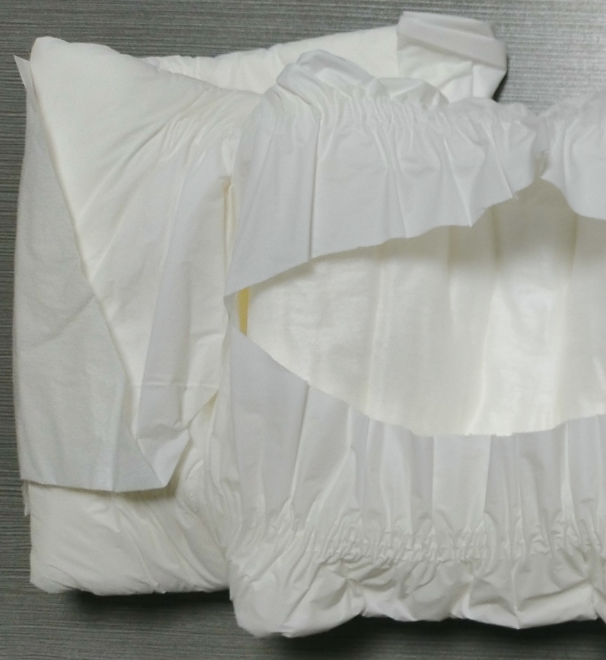

Supplement: S1 File — (ZIP) [file pone.0240285.s002.zip › Pictures of candicate homemade mask materials - ╕▒▒╛/11 Diaper.PNG]

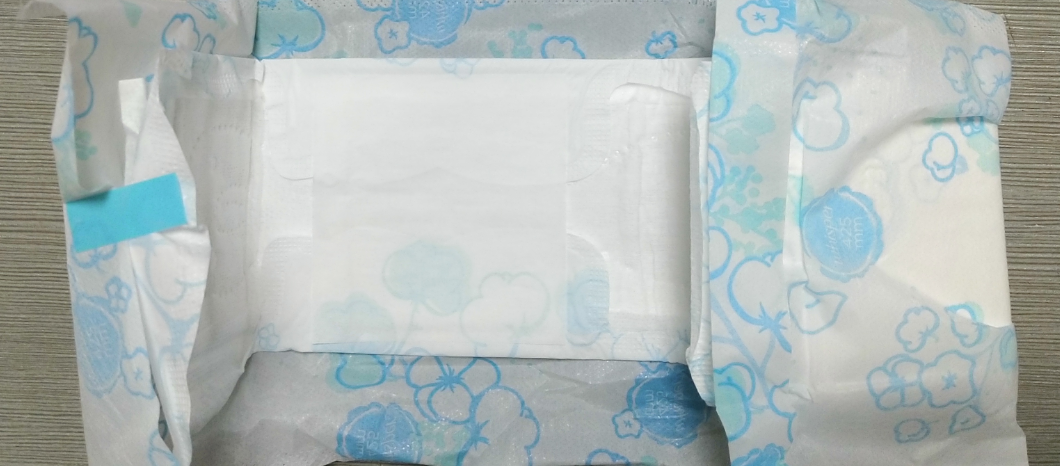

Supplement: S1 File — (ZIP) [file pone.0240285.s002.zip › Pictures of candicate homemade mask materials - ╕▒▒╛/12 Sanitary pad.PNG]

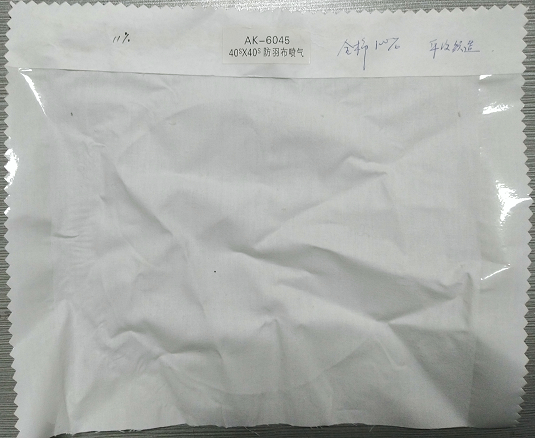

Supplement: S1 File — (ZIP) [file pone.0240285.s002.zip › Pictures of candicate homemade mask materials - ╕▒▒╛/13 Pillowcase A.PNG]

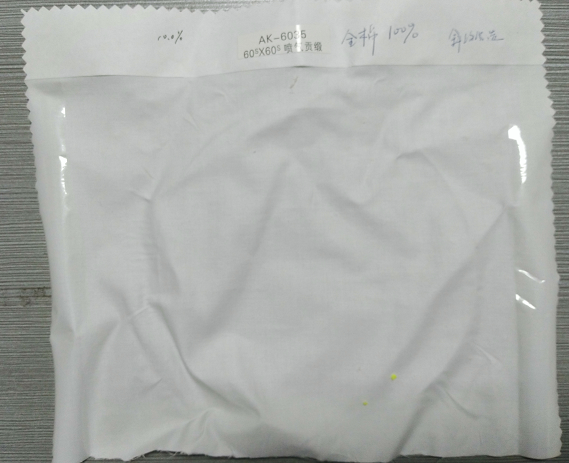

Supplement: S1 File — (ZIP) [file pone.0240285.s002.zip › Pictures of candicate homemade mask materials - ╕▒▒╛/14 Pillowcase B.PNG]

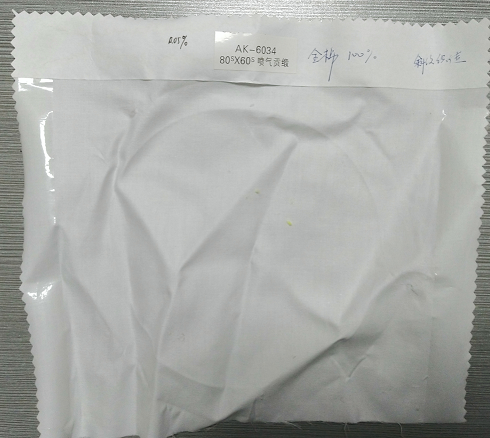

Supplement: S1 File — (ZIP) [file pone.0240285.s002.zip › Pictures of candicate homemade mask materials - ╕▒▒╛/15 Pillowcase C.PNG]

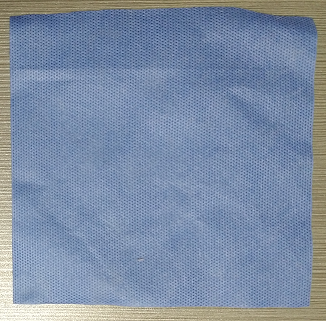

Supplement: S1 File — (ZIP) [file pone.0240285.s002.zip › Pictures of candicate homemade mask materials - ╕▒▒╛/16 Medical non-woven fabric.PNG]

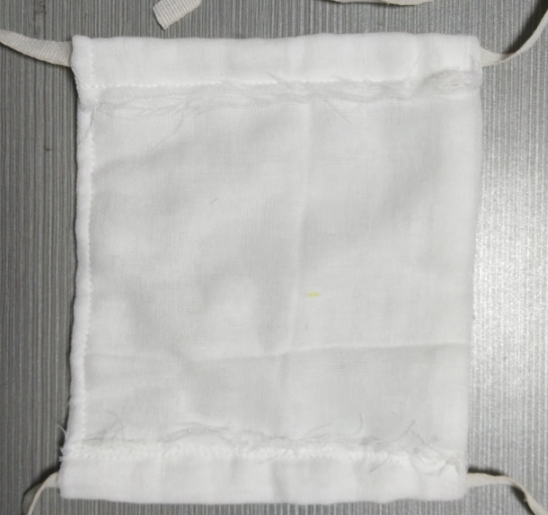

Supplement: S1 File — (ZIP) [file pone.0240285.s002.zip › Pictures of candicate homemade mask materials - ╕▒▒╛/17 Medical gauze.PNG]

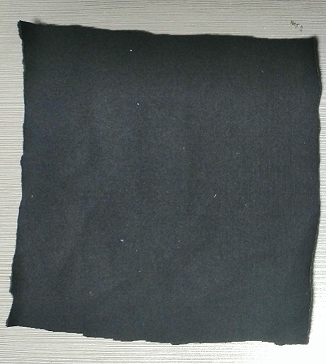

Supplement: S1 File — (ZIP) [file pone.0240285.s002.zip › Pictures of candicate homemade mask materials - ╕▒▒╛/2 Fleece sweater.PNG]

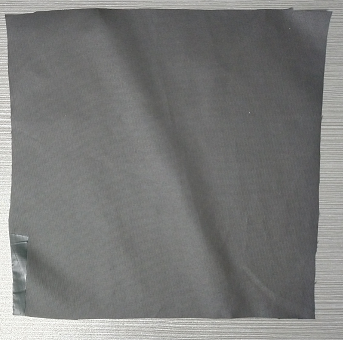

Supplement: S1 File — (ZIP) [file pone.0240285.s002.zip › Pictures of candicate homemade mask materials - ╕▒▒╛/3 Outdoor jacket.PNG]

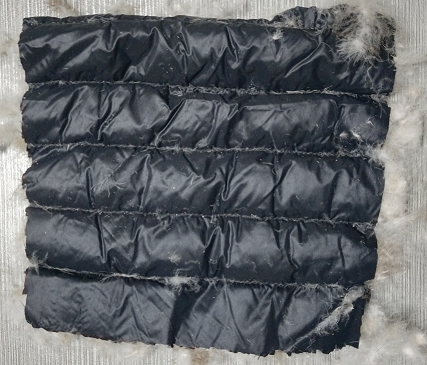

Supplement: S1 File — (ZIP) [file pone.0240285.s002.zip › Pictures of candicate homemade mask materials - ╕▒▒╛/4 Down jacket.PNG]

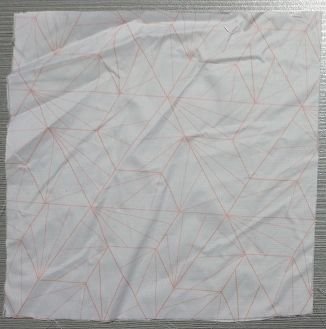

Supplement: S1 File — (ZIP) [file pone.0240285.s002.zip › Pictures of candicate homemade mask materials - ╕▒▒╛/5 Sun-protective clothing.PNG]

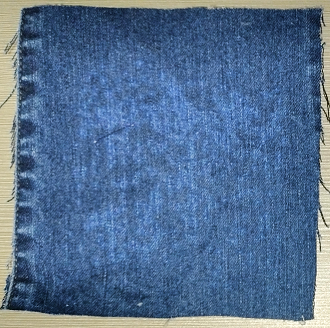

Supplement: S1 File — (ZIP) [file pone.0240285.s002.zip › Pictures of candicate homemade mask materials - ╕▒▒╛/6 Jeans.PNG]

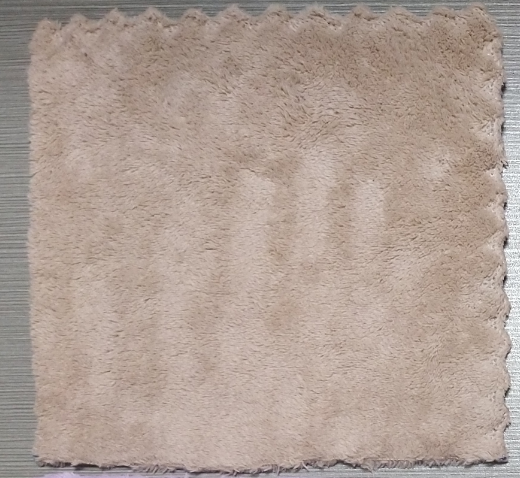

Supplement: S1 File — (ZIP) [file pone.0240285.s002.zip › Pictures of candicate homemade mask materials - ╕▒▒╛/7 Hairy tea towel.PNG]

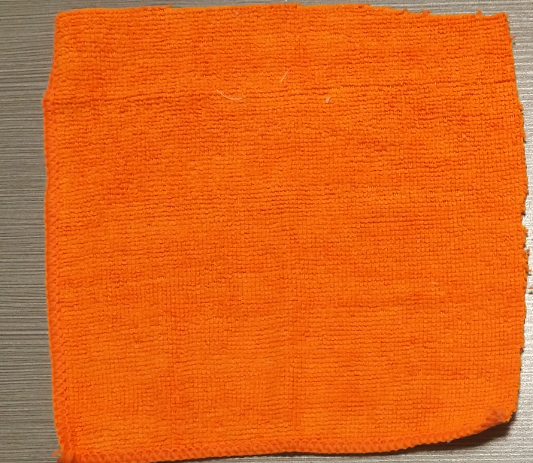

Supplement: S1 File — (ZIP) [file pone.0240285.s002.zip › Pictures of candicate homemade mask materials - ╕▒▒╛/8 Granular tea towel.PNG]

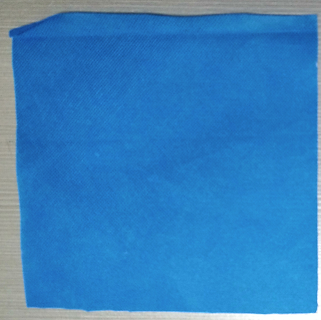

Supplement: S1 File — (ZIP) [file pone.0240285.s002.zip › Pictures of candicate homemade mask materials - ╕▒▒╛/9 Non-woven shopping bag.PNG]
